# Exploring barriers to clinical trial readiness among the myotonic dystrophy community: a mixed-methods study

**DOI:** 10.1186/s13023-026-04408-0

**Published:** 2026-06-18

**Authors:** Julia Stellmann Wrenn, Ali Ryan-Mosley, Jonah Watt, Erin Hellthaler, Tanya Stevenson, Andy Rohrwasser

**Affiliations:** 1 Third Plateau, Research & Evaluation Team, Miami, FL USA; 2 Third Plateau, Research & Evaluation Team, Brooklyn, NY USA; 3 Third Plateau, Strategy Team, Chicago, IL USA; 4 Third Plateau, Strategy Team, New Harbor, ME USA; 5 Myotonic Dystrophy Foundation, Oakland, CA USA; 6 663 Thirteenth Street, Suite 100, Oakland, CA 94612 USA

**Keywords:** Myotonic dystrophy, Clinical trial barriers, Mixed-methods, Patient advocacy groups, Registry, Rare disease

## Abstract

Myotonic dystrophy (DM) is a multisystemic disorder characterized by significant heterogeneity in symptom manifestation, progression, and severity. This variability complicates clinical trial design and implementation, thereby affecting therapeutic development. This study aimed to identify barriers to clinical trial readiness in DM and explore actionable solutions by incorporating perspectives from pharmaceutical partners, clinical trial site staff, people living with DM (PLDM), and caregivers. Focus groups, surveys, and interviews were conducted with pharmaceutical partners (*n* = 65), PLDM and caregivers (*n* = 35), principal investigators (*n* = 12), and clinical research coordinators (*n* = 12). Qualitative findings were thematically analyzed and triangulated with survey data to enhance validity. Three major barriers consistently emerged: (1) a lack of comprehensive, validated endpoints that capture outcomes meaningful to patients; (2) insufficient data sharing and coordination across industry; and (3) challenges in trial design, recruitment, and participant burden. PLDM emphasized under-studied but debilitating symptoms—such as dysphagia, fatigue, and cognitive impairment—that are rarely prioritized in clinical trials. Pharmaceutical partners cited regulatory uncertainty surrounding composite outcome measures as well as the value of quantitative myotonia indicators as informative holistic disease indicators. Limited access to natural history data, often prohibitively costly for smaller companies, reinforces competitive rather than collaborative dynamics. PLDM and caregivers also highlighted substantial financial, logistical, and accessibility challenges associated with trial participation. Participants favored several strategies to address these barriers: (1) convening a multi-stakeholder scientific session with the U.S. Food and Drug Administration (FDA) and establishing a working group to define and standardize patient-centered, clinically relevant endpoints; (2) developing a comprehensive, coordinated registry that integrates existing data sources, longitudinal health and study data, genetic diagnostic data, supports recruitment and refinement of study inclusion criteria, and enables post-market surveillance; (3) strengthening trial site preparedness and anticipating recruitment needs, including biomarker-driven cohorts; and (4) adopting patient-centered trial designs that minimize burden, with patient advocacy groups (PAGs) serving a central convening and advocacy role by bringing various groups together to discuss these topics. While barriers to DM clinical trial readiness are significant, they are addressable. Coordinated action among industry, regulators, clinicians, and advocacy groups—guided by patient priorities—will be essential. Implementing these strategies could accelerate therapeutic development, improve trial inclusivity, and ultimately enhance quality of life for individuals living with DM.

**Supplementary Information:**

The online version contains supplementary material available at https://doi.org/10.1186/s13023-026-04408-0.

## Introduction

Myotonic dystrophy (DM) is a rare, autosomal-dominant, multisystemic, and progressive disease affecting up to 1 in 2,100 people [1]. It is the most common form of muscular dystrophy in adults, and given its wide spectrum of severity, symptoms, and age of onset, is considered one of the most variable diseases in medicine [2–5]. Disease manifestations include myotonia, muscle weakness, fatigue, excessive daytime sleepiness, apathy, and cognitive difficulties. DM also frequently causes cardiac, gastrointestinal, vision, and endocrine complications [5]. As a repeat expansion disorder, DM demonstrates genetic anticipation, with disease severity worsening across successive generations [6, 7].

DM occurs in two distinct genetic forms: (1) Myotonic dystrophy type 1 or DM1, caused by a repeat expansion in the *DMPK* gene. DM1 is the more common type and can affect individuals of all ages. Subtypes include congenital DM (CDM), where symptoms present at birth and are linked to longer repeat lengths, and childhood/juvenile-onset DM, typically presenting before age 18 [8]. (2) Myotonic Dystrophy Type 2 or DM2 is caused by a repeat expansion in the *CNBP* gene, and it generally presents in adulthood [5, 9–11]. Emerging data suggest that repeat expansion disorders are shaped by a complex and evolving genetic architecture, including repeat size, sequence composition, modifier genes, and epigenetic features such as DNA methylation, which remains incompletely defined but may provide a foundation for more precise patient stratification and risk prediction [12–17].

The variability and multisystemic nature of DM present major challenges. For patients and caregivers, symptoms generate physical, cognitive, emotional, and logistical burdens [18]. Many caregivers are themselves affected, further complicating care dynamics. For clinicians, variability hinders timely recognition and diagnosis of the disease, and for those planning, designing and running clinical trials, it complicates guidance on meaningful selection of clinical trial endpoints. For the industry, it creates obstacles in trial design, patient stratification, and endpoint selection. For regulatory agencies, matching clinical trial endpoints with true and meaningful patient impact is a logical concern.

Currently, no disease-modifying therapies exist, and treatment remains limited to symptom management. Nonetheless, drug development efforts are progressing, indicated by a robust and expanding development pipeline, with more than 12 therapies in preclinical stages and over 10 in clinical trials [19]. Still, substantial barriers remain. Ensuring clinical trial readiness or the community overall being best prepared to conduct, engage, and succeed with clinical trials operations and outcomes requires coordinated action to address challenges faced by industry sponsors, trial sites, clinicians, patients, and families, while also anticipating barriers to treatment access once therapies become available.

Patient advocacy groups (PAGs) are central to this process. PAGs often serve as the first point of contact for disease-specific information and are recognized advocates for rare disease drug development. Yet DM’s rarity and variability complicate progress in drug development and clinical trials. Key challenges include small patient populations, insufficient natural history data, evolving regulatory frameworks, and difficulty defining consistent endpoints and biomarkers [20, 21]. Prior research has shown how muscular dystrophy PAGs have mitigated these hurdles. Huml et al. (2020) identified five effective strategies: establishing national registries, reducing barriers to patient participation, partnering with biopharmaceutical companies, collaborating with regulators on disease-specific guidance, and integrating market access considerations early in development. Huml et al. also emphasized the importance of direct patient engagement to identify needs, evaluate registries, and strengthen collaboration across stakeholders [21].

To accelerate progress toward effective therapies, continuous information exchange among patients, caregivers, clinicians, regulators, and industry is critical. People living with DM, as the true experts of their condition, must play a central role in shaping trial design and research priorities [22, 23].

This study builds on previous work by systematically assessing barriers to clinical trial readiness. The first phase captured challenges reported by industry partners, patients, and caregivers. These findings then informed further assessments with clinical and non-clinical trial site leaders. Together, results highlighted both organizational and systemic gaps, underscoring the need for targeted solutions and coordinated, ecosystem-wide action.

## Methods

To understand the gaps, barriers, and needs of DM community members and industry partners as they relate to clinical trial readiness, the following thematic questions were explored:

1. What are the current challenges and barriers to clinical trial readiness among industry partners, patients, the DM community, and the clinical trial sites?
2. What potential solutions to the challenges and barriers exist?
  - How do potential solutions align or not within and across these groups?
  - Which potential solutions are acceptable to the three groups?

We employed a sequential mixed-methods approach by gathering, analyzing, and reporting findings that included both qualitative and quantitative data. The qualitative data were collected first, then analyzed and used to inform the quantitative survey.

Each form of data collection included a consent process outlining confidentiality, data privacy, and potential harms of participation. This study was determined to be exempt by the Advarra Institutional Review Board (IRB) as Pro00081721.

### Qualitative data collection

Third Plateau directly engaged 75 individuals through 11 interviews and 15 focus groups, contacted through the Myotonic Dystrophy Foundation’s ecosystem network. These conversations were semi-structured, using guided prompts to elicit opinions and experiences in an exploratory and open-ended way. This process included:

- 11 focus groups with different pharmaceutical companies (40 participants);
- 11 individual interviews with people living with DM (PLDM);
- 4 caregiver focus groups (24 participants).

Pharmaceutical companies with active DM drug development programs were identified by MDF and invited to participate in focus groups. We intentionally invited companies that are in various stages, including discovery, preclinical, and clinical trial phases and that range from newer, smaller to larger pharmaceutical companies in order to explore a range of experiences.

PLDM were identified based on their participation in the MDF registry. PLDM with a history of participation in clinical trials for treatments or natural history studies, a range of disease severity, and various disease stages were invited to participate in interviews.

Caregivers were identified by MDF’s network of caregiver groups and invited to participate in focus groups. These focus groups were stratified by type of DM (DM1, DM2, Juvenile Onset, and CDM).

While MDF was involved in participant selection and outreach, data collection was conducted entirely by the authors from Third Plateau. This was intentional to reduce biased responses from participants who were familiar with MDF and those authors. Written and verbal consent were obtained for PLDM and caregivers, while verbal consent only was obtained for pharmaceutical partners due to privacy concerns.

### Quantitative data collection

We distributed two confidential surveys, one to pharmaceutical industry partners and another to representatives at current and potential interventional and/or natural history study sites. For simplicity, these sites are collectively referred to as Clinical Trial Sites, although they might not be currently running interventional drug trials. Written consent was included in the survey prior to any questions.

The Pharmaceutical Partners Questionnaire was distributed after each focus group with pharmaceutical partners, and 27 complete responses were received. Because participants were able to share the survey with other colleagues outside of the focus group and the survey was not required, we cannot calculate the response rate because we do not know the total distribution volume. Participants were not asked to report their names or the names of their companies. The purpose of this survey was to collect additional relevant information that we did not have time for in the focus group.

In the second phase of the study, the Clinical Trial & Study Site Survey was designed sequentially based on initial analysis of the qualitative data in order to test assumptions, feasibility, and relevance of barriers that arose in the qualitative data. The questions in the survey were a mix of structured (e.g., Likert scale, yes/no) and open-ended questions.

The survey consisted of two versions, one tailored to the clinical trial principal investigators (PIs) and another tailored to trial site research coordinators. Participants were invited to participate in the survey via email based on their role at current DM clinical trial sites and DM clinics, which may be selected as future clinical trial sites. All known sites were included in the sampling pool. The Clinical Trial Site Survey received 24 complete responses, including 12 from clinical research coordinators at the clinical trial sites and 12 from principal investigators (PIs) who are running DM studies and trials. This represents response rates of 16% and 41%, respectively. These responses represent many different sites, though not all unique.

### Qualitative analysis

The qualitative data were analyzed first, as insights from the qualitative data informed the design of the quantitative survey with Clinical Trial sites. During the preliminary analysis process, we conducted a rapid qualitative data analysis process that elevated the key themes and findings across interviews and focus groups. Once key themes were identified, the preliminary findings were used to conduct an in-depth analysis, coding each transcript using a mix of deductive and inductive codes. This process was conducted in NVivo (Lumivero, Denver, CO), a qualitative coding software. From this, a qualitative coding matrix was created to explore patterns within and among key themes and various participant groups. These findings, in addition to analysis of the Pharmaceutical Partners Questionnaire, guided and informed the design of the Clinical Trial Site Survey.

### Quantitative analysis

**Fig. 1:**
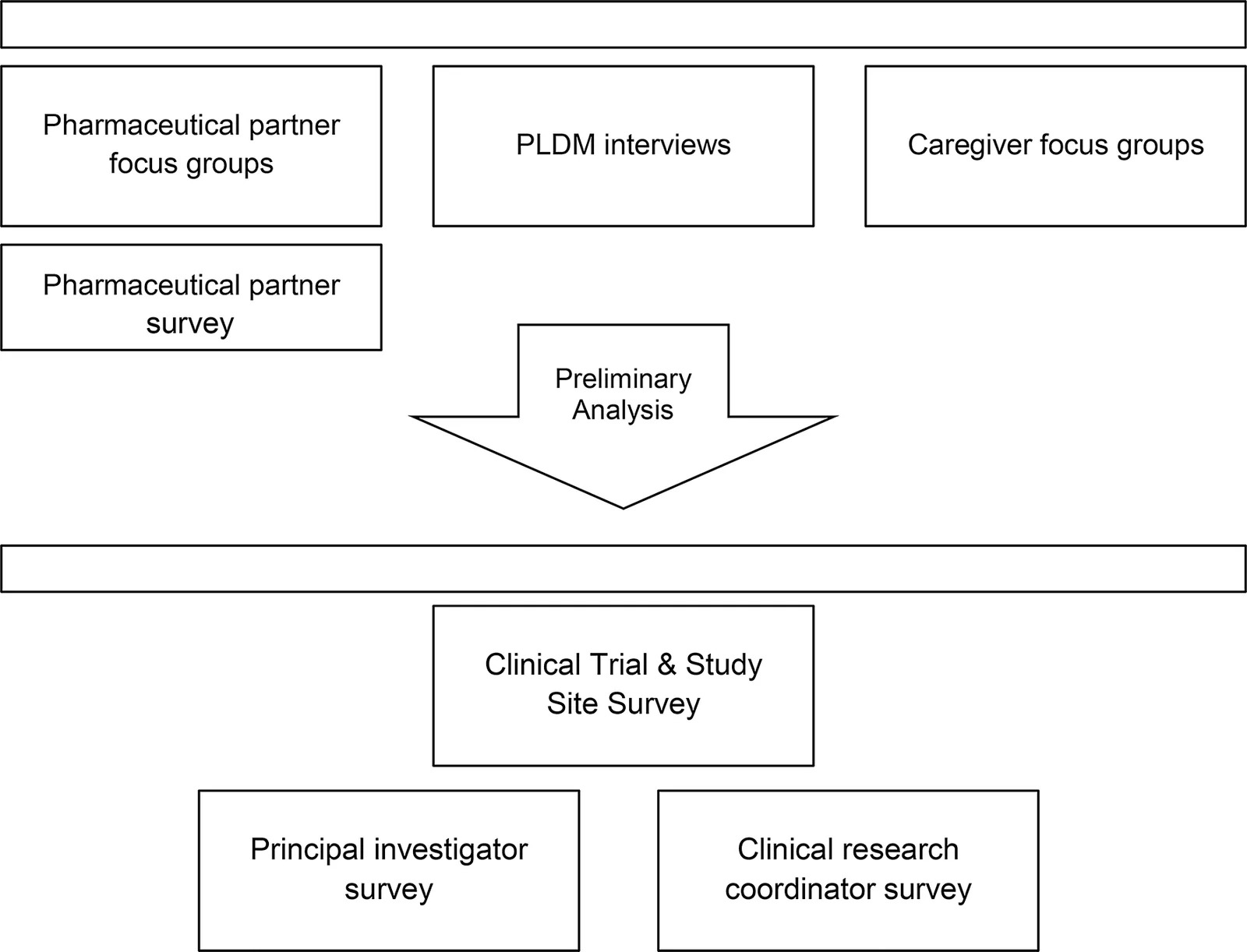
Phases of data collection for this mixed-methods study

The survey was distributed through Qualtrics (Qualtrics, Provo, UT), a survey distribution software. To analyze the quantitative survey data, both Qualtrics and Excel’s descriptive statistics tools were used. Survey responses were analyzed by role (PI or Coordinator), and treated as independent regardless of site location. The key themes and patterns from the qualitative data were analyzed first triangulated with data from the two surveys. Data from the surveys was given more depth and nuance by pairing it with individual perspectives from the focus groups and interviews. Additional context and information were provided. The mixed-methods process is outlined in Fig. 1.

## Results

Tables 1, 2 and 3 summarize participant characteristics. The study engaged four primary groups: pharmaceutical partners, people living with DM (PLDM), caregivers, and clinical trial site principal investigators (PIs) and research coordinators.

**Table 1:** Number of participants in interviews and focus groups by type of DM

Conversations with Patient and Caregiver Community			
Type of DM		Participants	Form of Involvement
DM1	DM1 Adults	17	Caregiver focus group (*n* = 7)
			Interviews with PLDM (*n* = 10)
	Juvenile-onset DM	8	Caregiver focus group (*n* = 8)
	Congenital DM	4	Caregiver focus group (*n* = 4)
DM2		6	Caregiver focus groups (*n* = 5)
			Interview with PLDM (*n* = 1)

**Table 2:** Breakdown of engagements with pharmaceutical partners

Engagement with Pharmaceutical Partners		
Method	Participants	Form of Involvement
Focus group	40	11 focus groups with 11 different companies
Pharmaceutical Partners Questionnaire	25	25 questionnaire responses from focus group participants

**Table 3:** Clinical trial site survey participants by role

Clinical Trial Site Survey Participants	
Survey Type	Number of Responses
Clinical Trial Site Research Coordinator Survey	12
Clinical Trial Site Principal Investigator Survey	12

Results from the thematic analysis of the qualitative data are reported as thematic findings based on the analysis. Three major categories of barriers to trial readiness emerged:

1. Lack of Comprehensive and Meaningful Endpoints.
  - Current Endpoints Do Not Reflect the Multi-system Nature of the Disease.
  - Importance of Patient Input for Endpoints.
  - Need for Further Research on Endpoints.

2. Industry Need for Data Sharing and Coordination.
  - Importance and Cost of Natural History Study Data.
  - Competition Instead of Collaboration.
  - Potential Solution of a Centralized Registry.

3. Challenges with Trial Design and Participation.
  - Prioritization of Known Participants in Recruitment while avoiding selection bias.
  - Issues with Finding and Enrolling in Trials.
  - Trial Accessibility and Burden of Participation.

**Fig. 2:**
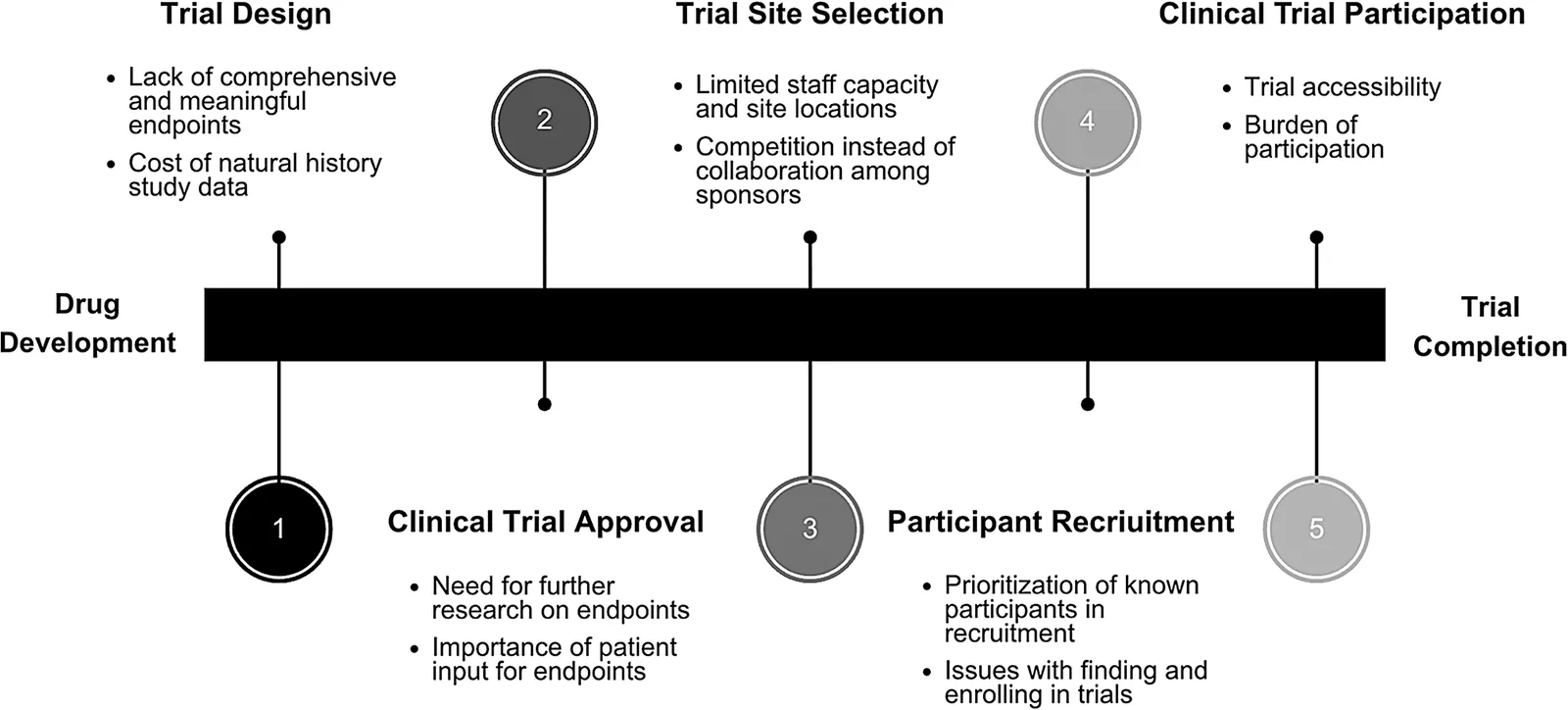
Roadmap of barriers to successful clinical trials for DM

Each theme is examined in detail below, with triangulation from the Clinical Trial Site Survey and Pharmaceutical Partners Questionnaire. Survey results are reported in aggregate to emphasize result patterns. Figure 2 maps and summarizes barriers along the trajectory of interventional trial design and execution.

### Lack of comprehensive and meaningful endpoints

#### Current endpoints do not reflect the multi-system nature of the disease

Across groups, participants emphasized the absence of standardized, validated, and patient-centered trial endpoints. DM’s hallmark symptom is myotonia, but the disorder affects multiple organ systems and varies widely, even within families. In most cases, PLDM and caregivers described years-long diagnostic delays due to heterogeneous symptoms and limited clinical awareness.

PIs and pharmaceutical partners acknowledged the challenge of controlling for disease variability in trial design, underscoring the need for endpoints that capture DM’s multisystemic presentation.

Most pharmaceutical partners reported incorporating Video Hand Opening Time (vHOT) in their designs, but noted high variability in both vHOT and other outcome measures. Several cited disagreements with regulators, particularly the FDA, regarding acceptable endpoints and what constitutes and defines meaningful change for patients.

*I don’t think we’re so interested in producing a statistically significant improvement in the relaxation in the muscle on the index finger on one hand*,* and saying we’ve got a successful drug. We’re trying to find methodologies to assess the whole of the phenotype*,* and we invested a massive amount to try and develop an outcome measure that didn’t work*,* to do that.*

*- Pharmaceutical Partner*

We’ve had long debates with the [regulatory agency] about composite outcome measures that allow you to have a measure of brain function, a measure of muscle function and then collapse them into one measure, and the [regulatory agency] won’t agree to that. So it’s difficult when you’ve got a population where almost any organ system of the body can be compromised, but isn’t necessarily compromised in any one patient, how do you come up with an outcome measure?

*- Pharmaceutical Partner*

Participants noted that these regulatory uncertainties make it difficult to identify which outcomes are meaningful, what endpoints might demonstrate improvement across DM’s diverse spectrum of symptoms, and whether an identified trial endpoint is truly a proxy for patients’ experiences and ultimately an indicator of meaningful change or improvement. This uncertainty reflects the fundamental issue of how to define “meaningful change” in clinical trial settings that also translates into real improvements in patients’ quality of life. Not surprisingly, this issue also transcends and manifests in the lack of regulatory clarity.

#### Importance of Patient Input for Endpoints

PLDM and caregivers valued vHOT but stressed the need for broader endpoints, including measures of GI function, cardiac function, cognitive decline, daytime sleepiness, and apathy. These symptoms profoundly affect daily life but are rarely assessed beyond questionnaires. Concerns extended to other muscles, such as cardiac and pharyngeal.

*There’s no biomarkers for speech or for swallowing. Swallowing is a big deal. I’ve choked four times. I needed the Heimlich three times*,* the fourth I did to myself. - PLDM*

*You know*,* there are muscle treatments*,* studies are going on*,* but the key is going to be getting a brain treatment. The executive function loss of kids needs to be reversed. Too many of them are robbed of their futures and their dreams because they just don’t have the executive functions. - PLDM*

Participants criticized patient-reported surveys as inconsistent, yet noted their regulatory endorsement.

*The [regulatory agency] has pointed us to certain patient reported outcomes*,* like the DM1-Activ. And then when you take that to adult onset patients*,* they hate it*,* and it’s a standard … You can see that the [regulatory agency] has been giving the same advice to all of the pharma companies in the space. And it’s now a running joke of the ‘soup questionnaire*,*’ because the first question is*,* can you eat soup? - Pharmaceutical Partner*.

#### Need for further research on endpoints

Less than half of pharmaceutical partners (45%) felt moderately ready to assess non-muscular endpoints; 20% reported being not very ready. 35% judged the broader field as not very or not at all ready, with cost and regulatory hurdles cited as key barriers. Survey data corroborated and confirmed these concerns: 90% of PIs agreed that more research is needed on endpoints, and 90% supported a structured forum to advance consensus.

*So this is where there is still a lot of discussion and also no agreement between the community and the [regulatory agency]…. We were quite convinced with our study design … yet we’ve confronted some*,* I don’t want to call it criticism*,* but further clarifications needed for what is clinically meaningful for these patients. If myotonia gets improved … What does it mean in terms of the disease progression? What does it mean in terms of patients’ perception of improved quality of life*,* and how should we measure it? So*,* there is a battery of validated tools as of today*,* and yet we hear different opinions of acceptance by the regulators*,* by [regulatory agency] … there is no consensus on that. So it makes it really difficult to talk about the study designs that would kind of accommodate all these different needs. - Pharmaceutical Partner*

### Industry need for data sharing and coordination

Natural history data, patient experience data, as well as–when available–genetic diagnostic data are considered foundational for the drug development process. In DM, the identification of comprehensive and meaningful endpoints is still limited and hindered by the fact that DM natural history and clinical trial data are not yet freely accessible. In general, participants identified the siloed and competitive culture among trial sponsors and research organizations as a deterrent to data sharing, trial design, and collaboration, and criticized the lack of a central, free database containing natural history data, disease progression data, and genetic information.

#### Importance and cost of natural history study data

Participants identified siloed data and a lack of coordination as barriers. Natural history data, while critical, is largely restricted to a “pay-to-play” model. The Establishing Biomarkers and Clinical Endpoints in Myotonic Dystrophy Type 1(END-DM1) study by the Myotonic Dystrophy Clinical Research Network (DMCRN) is the most comprehensive resource, but sponsorship is costly. The importance and pioneering impact of this study was widely recognized by the pharmaceutical participants, many of whom praised the study for filling a critical gap in natural history research on DM.

While the END-DM1 study has been enabled and its expansion funded by early entrants in the DM drug development space, these early funders receive regular “data-cuts” serving as reference points for general disease progression, prior to clearly outlined publication plans and milestones of the entire study. This transactional approach is perceived as a major barrier for some smaller pharmaceutical companies with limited budgets who are trying to enter the space. One participant remarked:

… I’ve never worked [on a disease] where you’ve had to pay. … I’ve worked in places where you have to pay, but it’s just to cover costs, it’s not to make money … I think other advocacy groups for rare diseases, they also charge based on the company’s size and revenue. There’s another way of doing it. And I think the DMCRN actually do not bother if you’re like a top 10 pharma or a small, tiny biotech, they charge you the same flat rate, which is kind of really, really uneven playing ground here.

*- Pharmaceutical Partner*

Some participants raised the lack of planned public dissemination of the full dataset, despite FDA and advocacy group funding for the study, as a potential concern. Because the END-DM1 study is so critical, pharmaceutical partners were eager for access to the data. Many pharmaceutical companies separately acknowledged a willingness to share their own data, but they cited competitive barriers and a general lack of sharing as the industry standard. Suggestions included advocacy groups convening sponsors to coordinate. Industry representatives pointed to opportunities for sharing placebo or failed trial data, noting success in ALS. Yet most stressed the need for collective agreements to reduce individual risk.

[Placebo data] is of great use to patient groups who are setting up a database establishing a natural history where you have such a frequent collection of data in a very systemic fashion … I would not want to give [data] to a patient group upfront unless I am pushed. And I’m not only me. If other sponsors are pushed too, I would say, ‘well, I’m happy to do it, would [company name] do it? Would [company name] do it, would [company name] do it? Would [company name] do it?’ And MDF tells them ‘look guys we want to set this consortium. This will be an anonymized data set but will support your … efforts if you commit to submission of this data.’

*- Pharmaceutical Partner*

Some large pharma partners expressed less concern about data sharing, viewing DM as relatively advanced in terms of available data. These pharma partners applauded the access to crucial natural history study data provided by the DMCRN in the END-DM1 study.

I think myotonic dystrophy is actually ahead, and that through the DMCRN, most companies do have access to that data. Do we pay for it? Of course we pay for it. But most of us do have access to a good chunk of reasonably well-collected natural history data.

*- Pharmaceutical Partner*

#### Competition instead of collaboration

Despite shared concerns, many companies reported siloed approaches. The lack of a standardized assay for *DMPK* gene repeat lengths was particularly noted, with larger firms developing in-house tools without wider dissemination.

*This is a disease caused by the presence of CUG repeat RNA*,* right? There is no assay for CUG repeat RNA*,* so … there is no way of measuring whether you’re treating the disease. That’s like trying to do HIV research without a way of measuring HIV virus or anti-infective research*,* without a way of measuring whether you can remove infections. And we’re having to build our own assay. And when I’ve asked all of the academics that we speak to*,* why is there no assay*,* I get no good answer. - Pharmaceutical Partner*

Pharmaceutical partners and PIs also expressed growing interest in genetic modifiers and CTG repeat length, though few current trials incorporate them. 80% of PIs judged repeat composition and modifier loci as likely to influence disease severity. While promising, this could complicate recruitment if the infrastructure for genetic stratification is lacking.

#### Potential solution of a centralized registry

When asked about registries, participants generally supported the idea of a centralized natural history registry, either through the coordination of existing registries or the development of a new resource. No such registry currently exists for DM, although there are several registries currently operating. Desired data include demographics, natural history data, natural history study data (e.g. END-DM1), comprehensive and inclusive clinical data from the patient’s electronic health record (EHR), genetic testing data, all clinical visit data, reference to available biospecimens, and consent for trial eligibility testing.

… Collecting [data on symptoms] and then constantly kind of looking in [the registry] and saying like, ‘what are the key symptoms’ so that you could eventually go to a physician and say ‘you see people with diarrhea, you see people with exhaustion, you see people with hand cramping. But if you see all three of those things together … you should think DM1.’

*- Pharmaceutical Partner*

Registries were also viewed as tools for patient education and awareness.

*Not every patient out there knows everything about their disease … so having the resources available to them is fantastic because that allows them to understand more about themselves*,* more about the opportunities that are open to them and their family. And that is where I think a patient registry becomes very helpful. So it’s not necessarily a recruitment tool … [but] the patients become more educated as well because they have outreach from other patient advocacy groups. - Pharmaceutical Partner*

60% of PIs (6 out of 10 participants) judged a comprehensive registry as helpful for trial planning, though fewer currently use registries for recruitment. This is likely due to the fact that most centers draw on their own pools of patients for study recruitment. Some participants cautioned against duplicative registries, stressing consolidation and core data sharing.

There already are other registries that are bigger, and what I don’t like to see … is all these little registries, and I think it’s better to have one central registry …. I don’t like rival registries. … It’s almost better if a lot of these organizations came together and just decided, ‘okay, well, we’re going to have one registry. We’re going to share core data.’ And then, yes, each [registry] will have the flexibility … [to] add other things depending on what they think they need. But it would be nice to have one core registry, or at least not the 100 that we have.

*- Pharmaceutical Partner*

Others expressed skepticism about the feasibility, citing reluctance to share and potential sensitivities around global access. Still, PLDM and caregivers were generally eager to contribute, though some raised concerns about privacy and insurance.

### Challenges with trial design and participation

For PLDM and caregivers, a major barrier to clinical trial readiness in the DM community is the actual design of trials and their ability to participate. Despite a willingness to volunteer for clinical trials, many participants discussed how the process of screening and being selected for trials requires much more than offering to be part of the study.

#### Prioritization of known participants in recruitment

Recruitment was perceived by PLDM and caregivers as heavily dependent on clinic affiliation. Many felt proximity to selected sites increased access, prompting some to travel interstate for care. Survey data confirmed that recruitment is largely through prior clinic or study connections rather than patient-initiated contact, with almost all PIs and research coordinators (90%, *n* = 10, of PIs and 89%, *n* = 9, of research coordinators) indicating that the participants are known from the clinic.

Furthermore, the majority responded that potential interventional trial participants are known from being registered in a “registry” (50% of PIs and 56% of research coordinators) or from a natural history study (50% of PIs and 44% of research coordinators, Table 4). The term registry here also implies “study record structures”. Patient-initiated contact in the clinical trial enrollment process played only a very minor role, cited by only 10% (*n* = 10) of responding PIs and less than a quarter of responding research coordinators (22%, *n* = 9).

**Table 4:** PI and research coordinator responses by answer choice. This data originates from the clinical trial & study site survey

Answer Choice (Participants Could Select Multiple Options)	PIs (*n* = 10)	Coordinators (*n* = 9)
*Participant is known from clinic*	90%	88.9%
*Participant is registered in a registry*	50%	55.6%
*Participant is known from other natural history study*	50%	44.4%
*Participant is known from END-DM1 natural history study*	40%	33.3%
*Participant initiated contact*	10%	22.2%
*Other (please describe)*	10%	11.1%
*None*	0%	0.0%

#### Issues with finding and enrolling in trials

Participants frequently noted difficulty identifying trial opportunities. PLDM described reliance on clinicaltrials.gov, which they found challenging to navigate. Consistent with a lack of interventional trials for juvenile and pediatric-onset disease forms as well as DM2, caregivers emphasized limited opportunities for DM2 and adults with juvenile-onset DM. Many PLDM reported educating their own physicians about DM, raising concerns about clinicians’ capacity to inform patients of trial options.

*It’s not a disease that … I would say a lot of doctors are familiar with. And so there’s a lot of misinformation or just no information. Like my dad*,* when he first was diagnosed*,* his doctor was like ‘oh well*,* you’re like his repeats are low*,* so you’re not really going to have any other symptoms.’ And then he had to have an emergency pacemaker placed in. And he would have died. … I’m very confident in the team of doctors I have*,* but I also have a wealth of medical knowledge to fall back on*,* whereas I think a lot of people don’t. And so*,* you know … You try to trust your doctor*,* but if your doctor isn’t putting in any effort to understand your disease then … They’re not really doing their job. - PLDM*

Both PLDM and caregivers requested more proactive dissemination of trial opportunities from advocacy groups.

*I think just helping to … make us more aware of what’s out there*,* at least until we get to a point where maybe we’re stable enough that we can take the time to do that. I don’t know if and when that’ll happen. But it would be nice to know*,* to be alerted of those types of things. And if there’s something that we’re interested in*,* we can research it more. But*,* you know*,* something that’s just say*,* ‘hey*,* you know*,* this is what’s out there.’ - Caregiver*

Participants also highlighted frustrations with trial pacing and regulatory delays. Pharmaceutical partners expressed interest in discussing accelerated approval pathways with regulatory agencies, like the FDA.

So from a regulatory policy standpoint, I think the [regulatory agency] has been very open to defining and discussing, I guess, accelerated approval pathways for other neuromuscular disorders. So I think it’s important to have a discussion with them around, what is the path to accelerated approval for myotonic dystrophy and that it should be a priority for them, you know? And how do we get to a place where the [regulatory agency] is having a discussion with companies right in terms of, are they willing to think about this as a regulatory pathway for myotonic dystrophy drugs?

*- Pharmaceutical Partner*

Exclusion criteria were another major concern. PLDM reported being excluded for DM-related comorbidities, age, or concomitant medications, which they perceived as contradictory. While such perception indicates a lack of understanding of the trial process per se, it also highlights education opportunities.

*When they’re looking for individuals with certain symptoms*,* finding the individuals within DM that don’t have other symptoms that are very characteristic of the disease. That’s very hard. So it really limits that pool of individuals that they have to draw from. - PLDM*

*Some of [the trials] you have to be off your meds. And if I didn’t take my Ritalin and my Nuvigil this morning*,* I wouldn’t be sitting up here talking to you. I’d be a dish rag. I was in one [study] where I had to be off them for I think it was two weeks. And I was still working full time. And it was hard. I had a lot of caffeine to get me through the day … So a lot of people have to be off certain meds they can’t really function without. So that’s a big issue I have. - PLDM*

PLDM participants also expressed disappointment with the screening process for trial participation. Disappointed when finding out they are excluded, PLDM felt the inclusion/exclusion criteria were at times conservative, and the screening process unclear and burdensome.

*So far*,* I haven’t [participated in a trial]. I did screen for one clinical trial. But … I didn’t fit the criteria because one of my blood works was off. So I did spend a whole day at [site] screening for the [company] clinical trial. It’s a phase three clinical trial. I would have loved to take part*,* but I didn’t qualify. - PLDM*

#### Trial accessibility and burden of participation

For clinical trial sites, staffing emerged as the top barrier (Table 5).

**Table 5:** Top 1–3 challenges in getting clinical trials for myotonic dystrophy up and running, according to PIs and Research Coordinators. The data originate from the clinical trial & study site survey

Answer Option	PIs (*n* = 10)	Coordinators (*n* = 9)
Staffing (number of staff)	70.0%	44.4%
Extensiveness of the assessment protocols	30.0%	33.3%
Navigating regulations/requirements	30.0%	44.4%
Finances	30.0%	11.1%
Other (please describe)	30.0%	11.1%
Competing with other neuromuscular disease clinical trials	20.0%	22.2%
Communication with sponsors	10.0%	11.1%
Equipment (e.g., purchasing, set up, function)	10.0%	0.0%
Participant retention	10.0%	0.0%
Participant enrollment	10.0%	0.0%
Participant recruitment	10.0%	11.1%
Staffing (staff expertise)	10.0%	11.1%
Participant education	0.0%	0.0%
Belief in the success of the intervention	0.0%	0.0%
Unsure	0.0%	33.3%

Interestingly and in contrast, 50% (5 of 10 respondents) of PIs also indicated that their site was prepared to start three or more trials in the next year, and 40% (4 of 10 respondents) indicated that they were prepared for 1–2 trials to start in the next year. With surveying sites that were and were not currently conducting interventional trials, staffing shortages point towards a potential bottleneck in trial execution, especially at a time when we expect more trials for DM to begin.

These constraints also point towards future bottlenecks in treatment rollout. Several therapies currently in late-stage development are infusion-based, placing additional demands on site infrastructure.

Across surveyed sites, all respondents (9/9 coordinators and 10/10 PIs) reported that their site is prepared to provide infusions for future studies or treatments. However, reported treatment capacity varied considerably. Most PIs (6/10) indicated they could treat 2–5 patients per week, one reported capacity for 1 patient per week, one for 6–10 patients per week, and two were unsure of their capacity.

When asked about the primary barriers to infusion-based treatment rollout, PIs most frequently identified time (6/10), followed by space (4/10) and personnel (4/10).

Taken together, these findings suggest that while baseline capability for infusion delivery exists, scalable capacity remains limited, reinforcing the potential for operational bottlenecks as therapies move toward approval and broader implementation.

PLDM participants highlighted perceived variable site readiness.

*What we’ve learned from all this is all centers aren’t created equal. Some have great infrastructure. Some have great admin*,* some have great coordinators. And others are missing that piece or that center doesn’t have the support of their own faculty to cheer you on and say*,* okay*,* let’s go. -Caregiver*

The logistical burden of remembering appointments and appointment details is high and particularly challenging for PLDM who experience brain fog, anosognosia, apathy, and fatigue. Clinical trial site research coordinators flagged this issue, some even naming it as the greatest challenge for participants in an interventional trial. Site research coordinators unanimously (100%, *n* = 9) indicated that they currently send multiple follow-ups and reminders about appointments. The symptoms of DM uniquely require this type of extra attention and follow-up for retention, even though PLDM are willing to participate in trials.

However, caregivers and PLDM alike noted that most of the logistical burden still falls on the caregiver, who might be taking care of multiple family members with DM and some of whom might also have DM.

PLDM and caregivers frequently described burdens of travel, site accessibility, and cognitive/financial stress.

*One of the things that gets talked about in our group a lot is just the actual physical hands-on challenges of flying. Getting on and off of a plane*,* the amount of equipment that it takes just to go to a hotel for a night because you have to have a certain level of surface to get off of for the bed*,* for the toilet*,* for all those things. - Caregiver*

The travel burden is high for many clinical trials, which might require multiple days of travel for relatively simple and quick but frequently occurring procedures, like a blood sample collection.

*We’re currently traveling … to be in a study. And it is really tiring when you go for a five-minute blood draw. And then you go home. You know*,* because [closer study site] wasn’t up and running yet. And it just was months and months and months of waiting. - Caregiver*

Financial barriers include upfront costs and delayed reimbursement, as well as reimbursements in less usable formats, like a prepaid debit card.

DM1 is associated with difficulty keeping a job. There’s a lot of poverty there. So if you’re on your own, it would be impossible to participate. Really, if you barely have enough money for food and rent, you can’t necessarily pay for something and wait a month to get it back. If you’re even going to get it back because maybe the systems are just too complicated to follow … And you can’t pay rent with a charge card … So there’s probably a lot of people who just can’t do it.

*- PLDM*

Caregivers described the compounded burden of supporting multiple affected relatives. The logistical burden of being in a study also emerged as a barrier to participation. Frequently, the time commitment to participate is not limited to the person participating but extends to caregivers or even whole families, as they may have to travel together. Some caregivers also discussed the burden placed on them due to the cognitive impairment of the people they care for.

*The obstacle to getting any of [my family members] into clinical trials is that I would be the one doing it because they don’t have the executive function to follow through and be looking to think to themselves*,* ‘oh*,* let me initiate this task*,* oh*,* let me maintain this task. Oh*,* let me finish this task.’ None of those skills exist for any of them. And so I would be the one doing it and I’m the one doing everything. - Caregiver*

*And in my case*,* my wife is dealing with all of it*,* and the follow-up and everything. If I was single I would not be able to handle it. …Because of the fatigue you get from DM1 and then you get a lot of cognitive issues*,* brain fog*,* inability to focus for long periods of time. - PLDM*

Despite the concerns from the perspective of patients and caregivers, few pharmaceutical partners or PIs see participant recruitment as a current barrier to clinical trial success, with most indicating that study recruitment is going really well. According to the Clinical Trial Site Survey, 80% (*n* = 10) of PIs agree or strongly agree with the statement, “patient recruitment for upcoming clinical drug trials is not a concern–we have enough participants that meet eligibility criteria,” and the other two responded neutrally. However, when asked about future levels of recruitment, multiple pharmaceutical partners expressed concerns, particularly considering the number of upcoming trials.

*We have lots of interest and lots of potential patients*,* so I think we’re feeling as of now*,* generally pretty good about [patient recruitment]. That may*,* of course*,* change as more studies start in the area. But right now*,* finding participants has not been the problem. We will see as more and more studies get up and going*,* particularly larger ones. - Pharmaceutical Partner*

## Discussion

### Barriers and potential solutions

This study identified three major barriers to clinical trial readiness in DM, reported consistently by pharmaceutical partners, people living with DM (PLDM), caregivers, and clinical trial site staff:

1. Lack of comprehensive and meaningful endpoints;
2. Insufficient data sharing and coordination across the industry; and;
3. Challenges in trial design, recruitment, and site and participant burden.

**Fig. 3:**
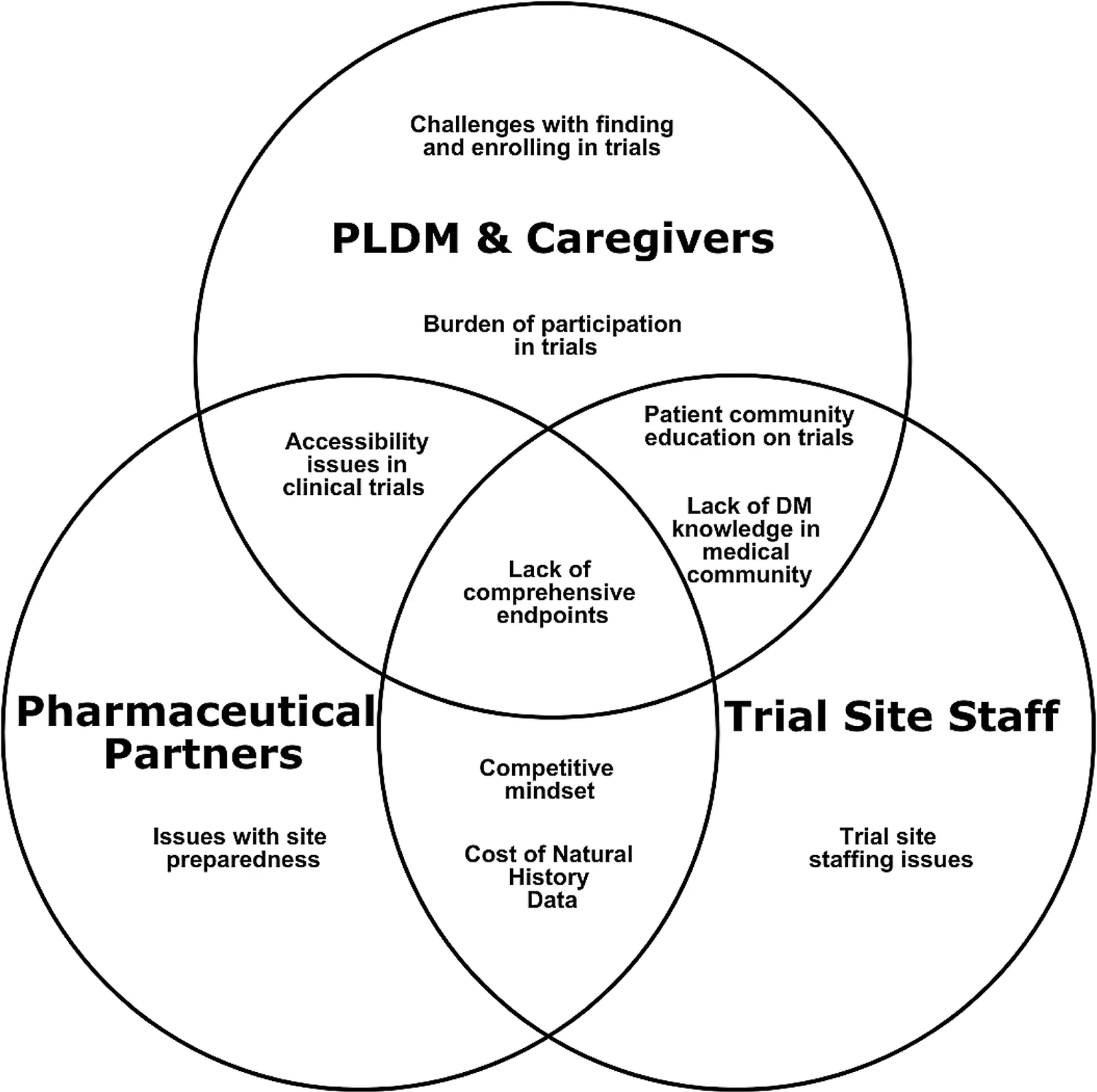
Primary areas of concern for each group of participants

Figure 3 provides a visual summary of the primary concerns identified by each participant group. Although all groups discussed every barrier, the figure highlights only the most frequent concerns. Primary concerns were determined based on data richness, frequency with which participants raised them, and the level of consensus across groups.

These findings align with prior landscape assessment research by the Myotonic Dystrophy Foundation (MDF) [24] and build on it by incorporating PLDM and caregiver perspectives. Our findings also echo other research exploring the role of patient advocacy groups for rare disease research [21].

**Fig. 4:**
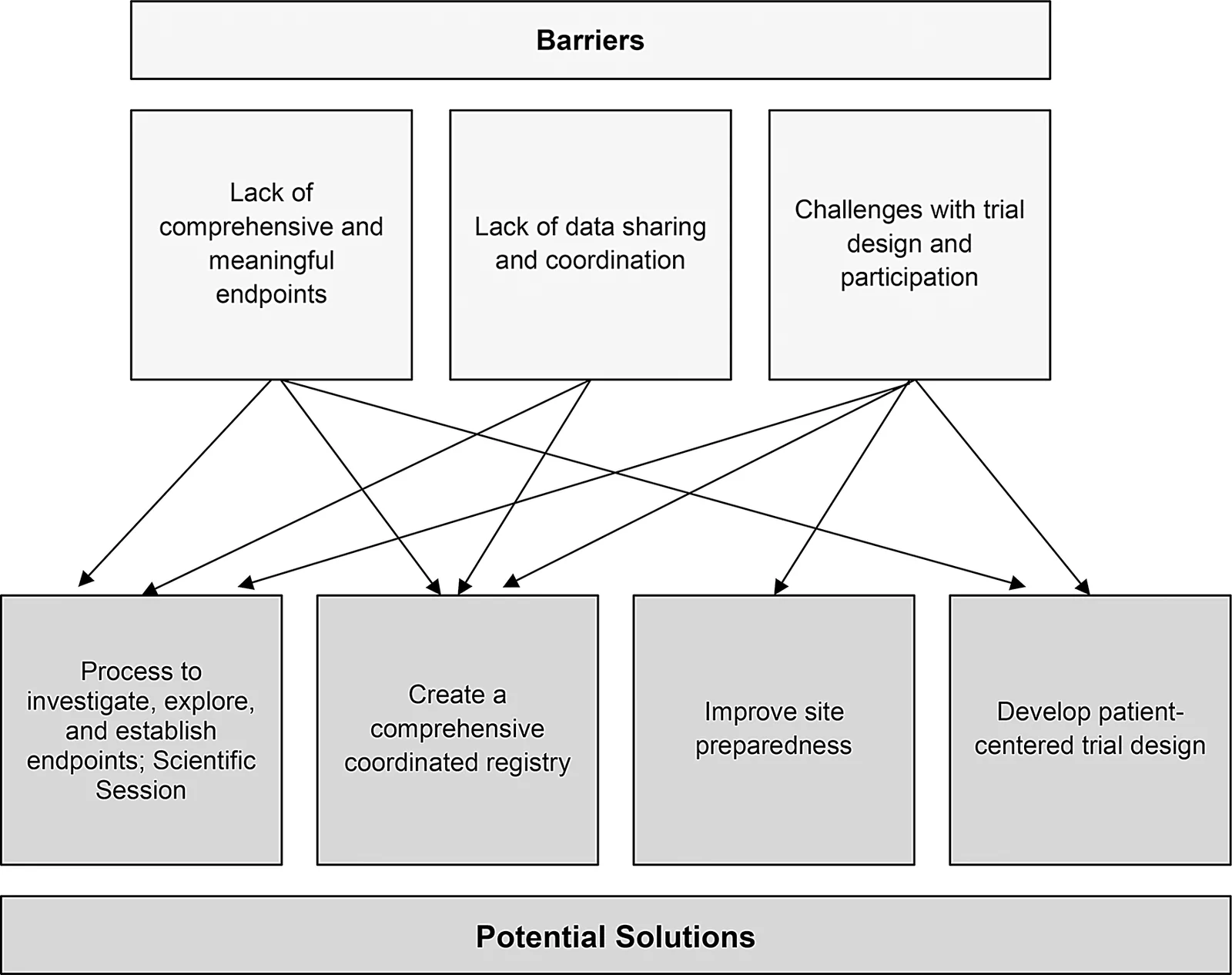
Potential solutions to identified barriers

The study also highlights potential solutions to address these barriers and move the DM community closer to effective treatments. Figure 4 illustrates these solutions.

### Exploring solutions

#### A process to investigate, explore, and establish endpoints; hosting a scientific session

Developing treatments is impossible without standardized, validated endpoints that reflect outcomes meaningful to patients. Current measures like vHOT capture hand myotonia but fail to represent the full DM experience. To establish a broader spectrum of informative endpoints that capture the patient experience within the relatively short duration of interventional studies, stakeholders emphasized the need for a scientific session with the FDA and a dedicated working group to establish multiple clinically relevant, patient-centered endpoints.

Action steps include:

- Hosting a multi-stakeholder session with the FDA (i.e., patients, caregivers, pharma, researchers).
- Expanding research into outcomes meaningful to patients (e.g., GI, cognition, fatigue).
- Exploring accelerated approval pathways.
- Promoting industry collaboration and open data sharing.

A strong alliance across all stakeholders, including regulatory agencies, would create continuous processes to find consensus, integrate advances in knowledge, and support targeted efforts to overcome delays. Most importantly, such concerted efforts could also positively influence reimbursement decisions for DM at large or for defined subgroups. PAGs, as trusted conveners, can ensure patient perspectives remain central in convening such forums and defining endpoints.

A central barrier remains the lack of knowledge and consensus regarding how to assess, measure and prioritize the full spectrum of DM manifestations. PLDM and caregivers stressed that outcomes most relevant to daily functioning, such as swallowing safety, fatigue, and executive function, are rarely prioritized. Pharmaceutical partners also reported persistent uncertainty regarding regulatory acceptance of limited (e.g. myotonia specific) but quantifiable endpoints and their generalizability capturing or reflecting broad disease impact and improvement thereof. As one partner noted,

We invested a massive amount to try and develop an outcome measure that didn’t work, and we are still struggling with how to demonstrate what matters to patients in a way that regulators will accept.

Endpoint development must be dynamic, not static. Iterative processes that incorporate emerging evidence, patient-reported outcomes, biomarker discovery, and lessons from ongoing trials are needed. Standing multi-stakeholder groups with regulators, advocacy organizations, and experts would enable continuous refinement of endpoints in real time. Such dynamic processes are essential to move beyond reliance on limited measures like vHOT and toward tools that reflect the heterogeneity of DM. They would also facilitate the adoption of innovative digital endpoints, wearable technologies, and advanced imaging modalities. Expanding knowledge in a structured but flexible manner would accelerate consensus and improve regulatory acceptance, while aligning trial outcomes with what PLDM and caregivers identify as meaningful improvements in quality of life.

#### Create a comprehensive, coordinated registry

Clinical trial progress is slowed by fragmented data and limited sharing. Competition among companies has created barriers to collaboration, and access to natural history data is particularly challenging for smaller firms.

A comprehensive data sharing platform would go beyond registry concepts and definitions. A comprehensive data sharing platform would include data from contact registries, natural history studies, completed clinical trials data, comprehensive clinical records, clinical trial data, patient reported outcomes and genetic testing data. Such longitudinal data structures would:

- Consolidate and represent data from existing registries and standardize practices globally.
- Support trial design, recruitment, and patient notification.
- Enable pre-screening for inclusion/exclusion criteria, reducing patient burden.
- Directly and indirectly capture meaningful patient impact.
- Allow sharing of placebo arm data to reduce participant exposure.
- Inform and guide the clinical care community.

Such platforms could support the definition of inclusion criteria, enable more efficient trial designs, support post-market surveillance, and lay the foundation for newborn screening. PAGs can play a central role in fostering collaboration, establishing best practices, and advocating for scalable data-sharing models that balance industry needs with societal benefit.

Integrated registries will be increasingly needed to support future clinical trials. Today, clinical trial recruitment in DM relies primarily on clinical care centers, with existing natural history studies serving largely as screening tools. However, this approach will not be sufficient for the next generation of therapeutic development [25–27]. As in many other disease areas [28], advancing drug development in DM will likely require more refined inclusion criteria that integrate genetic information, emerging modifier loci, clinical data, and patient-reported outcomes. Evidence for genotype–phenotype correlations and genetic modifiers of repeat instability is steadily increasing, underscoring the likely need for robust, well-curated datasets to define clinically meaningful patient subgroups [15, 17, 29–31]. Furthermore, experience from other rare diseases demonstrates that disease-modifying therapies are most effective when administered early, before irreversible manifestations occur [28]. As the field moves toward earlier intervention or relies on more comprehensive inclusion criteria, recruitment strategies based solely on local clinical networks will no longer suffice [32]. Comprehensive, longitudinal patient registries will be essential to identify appropriate cohorts rapidly and to enable timely therapeutic development and intervention [33, 34].

Projecting pre- and post-market approval needs, a culture of collaboration, and a comprehensive registry that benefits industry, researchers, and PLDM is paramount. PAGs, as trusted partners, are uniquely positioned to promote this collaboration and support the development of a registry or the integration of existing ones.

#### Improve site preparedness and recruitment

Recruitment is not currently viewed as a critical issue, but with multiple trials advancing for DM, along with trials for other diseases, demand might soon outpace current capacity. Many sites rely on existing patients for enrollment and face staffing shortages that could limit participant support. Furthermore, if current interventional trials prove inconclusive or require refined genetic inclusion criteria, current participant pools may prove insufficient.

To prepare and to position towards therapy delivery in the post-approval future, trial sites and sponsors should coordinate to:

- Plan and invest in staffing and research coordinator support.
- Invest in space and training for infusion-based treatments.
- Anticipate biomarker-based, targeted trials, which may reduce sample sizes but require broader registry data.
- Draw lessons from spinal muscular atrophy (SMA), where rigorously defined cohorts enabled efficient trial success [35, 36].

Early preparation is key to ensuring readiness as more DM trials move forward and also as treatments are approved and ready to administer. Many current treatment candidates are infusion-based, and our research shows that few trial sites are ready to deliver infusion-based treatments at scale. Sites need to be preparing now for additional trials and for starting to deliver treatments to PLDM.

#### Develop patient-centered trial design

PLDM and caregivers repeatedly underscored the heavy burden of trial participation, including financial, logistical, and physical demands. Unless trial design is more patient-centered, participation will skew toward wealthier or less burdened individuals, limiting inclusivity and potentially biasing results.

Recommendations include:

- Covering travel, hotel, and childcare costs upfront.
- Offering flexible reimbursement methods (direct deposit, cash, check).
- Providing at-home visits for blood draws and basic testing.
- Training staff in DM-specific care needs.
- Expanding opportunities for patients to initiate contact with trial sites.

As one PLDM explained, *“Failure to address inequities may lead to skewed samples of healthier*,* wealthier participants.”*

Importantly, PAGs have a central role in this area. By elevating patient voices and working with sponsors, they can advocate for participant-friendly trial structures, ensure adequate financial and logistical support, and help design approaches that reduce barriers for PLDM and caregivers. They can also monitor whether trial practices align with patient priorities and push for changes when gaps remain.

### Integrated approach

These recommendations are inherently interdependent. Validated endpoints rely on robust and harmonized data; registries support both patient identification and trial design; and patient-centered approaches depend on adequate site capacity and infrastructure. Patient advocacy groups (PAGs) are uniquely positioned to integrate these efforts by convening stakeholders, amplifying patient perspectives, and promoting alignment across the ecosystem. Addressing these barriers in a coordinated manner will be essential to strengthening trial readiness and accelerating therapeutic development in myotonic dystrophy (DM). Figure 4 provides a roadmap linking these solutions to the barriers they are intended to address.

Several limitations of this study should be acknowledged. First, the use of purposive sampling introduces potential selection bias. Survey responses from research coordinators, principal investigators (PIs), and pharmaceutical partners were limited in number and may not fully represent the global landscape, particularly given the relatively early stage of DM1 clinical trial activity. Although outreach was conducted internationally, most respondents were U.S.-based. Second, people living with DM (PLDM) and caregivers engaged through MDF may be more connected to advocacy networks than the broader patient population, potentially influencing perspectives on trial readiness and awareness. Third, reliance on self-reported data from surveys and interviews may introduce social desirability bias. Fourth, this study primarily reflects the DM1 landscape. Given the current focus of therapeutic development on DM1, representation of DM2 participants was limited, which constrains the generalizability of these findings to DM2.

Importantly, the challenges and opportunities identified in this study reflect stakeholder-derived perceptions within a rapidly evolving research ecosystem. Many of these barriers represent structural and operational constraints that are common across rare disease drug development, rather than limitations of specific initiatives. The findings and proposed actions are intended to complement, strengthen, and build upon existing efforts, including the END-DM1 study and the broader DM-CRN, to further enhance clinical trial readiness and accelerate progress toward effective therapies. Further research is needed in many areas, including meaningful endpoints, the integration of genetic determinants (repeat size and composition, genetic modifiers and methylation status), and preparations for the delivery of infusion based therapies.

In conclusion, our findings highlight an urgent need for the DM community to align around the development of comprehensive, standardized, and clinically meaningful endpoints that capture the multisystem nature of the disease. Equally important are strengthened frameworks for data sharing, improved coordination across stakeholders, and the systematic incorporation of patient and caregiver perspectives to reduce participation burden and improve trial accessibility.

We propose several actionable priorities: establishment of a dedicated endpoint working group, convening of a scientific session with the U.S. Food and Drug Administration (FDA), development of a comprehensive and accessible registry, strengthening of trial site readiness, and broader adoption of patient-centered trial designs. At the same time, further research is needed in endpoint validation and the inclusion of genetic factors (repeat size and composition, genetic modifiers and methylation status) and the operational requirements for scaling treatment delivery, particularly for infusion-based therapies.

With multiple therapeutic candidates advancing through development, the field is at a critical inflection point. Building the necessary infrastructure, aligning stakeholders, and embedding patient priorities into trial design will be essential to accelerating the path to effective therapies and improving outcomes for individuals living with DM.

## Supplementary Information

Below is the link to the electronic supplementary material.

Supplementary Material 1

## Data Availability

The datasets generated and/or analyzed during the current study are not publicly available due to confidentiality of the participants but are available from the corresponding author on reasonable request.
