# Supplementary material for: Exploring barriers to clinical trial readiness among the myotonic dystrophy community: a mixed-methods study
Source: Orphanet J Rare Dis. 2026 Jun 18;21:329. doi: 10.1186/s13023-026-04408-0 (PMC13602584; doi:10.1186/s13023-026-04408-0)
Supplement: Supplementary file 1 [file 13023_2026_4408_MOESM1_ESM.docx]

**Appendix: Guide and Survey Questions**

**PLDM Interview Guide**

1. Can you tell me a little about yourself and share how you got connected to MDF?
2. Have you participated in a natural history study?
   a. If yes, can you please discuss what that process was like for you?
      i. What went well?
      ii. What would you like to see changed?
   b. If not, why not?
3. Do you feel like you would sign up for a natural history study today if offered the chance?
   a. Why/why not?
   b. Is there something that would make you sign up immediately?
   c. Is there a reason you potentially hesitate?
4. What are the barriers, if any, for you personally to enroll in a natural history study?
5. Have you participated in any interventional (drug) clinical trials in the past?
   a. If yes, what was that experience like for you?
      i. Did you face any challenges when enrolling in this trial?
      ii. Was there a concierge service to help you with travel and logistics to the trial appointments?
      iii. Were there any related financial costs to participating that you had to cover yourself?
      iv. Did you face any challenges completing the trial? Please describe.
   b. If not, are there any particular reasons why you have not?
6. Do you feel like you would sign up for a clinical drug trial today if offered the chance?
   a. Why/why not?
   b. Is there something that would make you sign up immediately?
   c. Is there a reason you potentially hesitate?
   d. What are the barriers, if any, for you personally to enroll in a clinical trial?
7. In an ideal world, what would it look like for you to sign up and participate in a clinical trial?
8. Do you participate in a registry, for example MDF’s Family Registry or the National Registry at the University of Rochester?
   a. If yes, why?
      i. What are any benefits you see in participating?
      ii. What are any challenges/barriers you see in participating?
   b. If no, why not?
      i. Probe for benefits
      ii. Probe for barriers
9. Some clinical trial sites also run natural history studies, so they also have access to information about those participants. They may recruit people form their natural history study who meet the trial inclusion criteria. Are you aware of the connection between being in a natural history study and being enrolled in a clinical drug trial?
   a. Does knowing this information make you more or less willing to participate in a natural history study?
   b. Does knowing this information make you more or less willing to sign up for a patient registry that already includes your natural history data?
10. Comprehensive registries containing information about diagnosis, surveys and assessments completed directly by patients, as well as personal health information and records could greatly improve clinical trials by helping with better participant recruitment and matching. MDF is interested in designing and managing a new comprehensive registry. What comes to mind for you in thinking about participating in a registry that has all of this information about you?
    a. Would it be beneficial for your healthcare provider to provide or enter your clinical health data into a registry FOLLOWING your permission and consent?
       i. Why/why not?
       ii. Probe for data entry burdens
    b. Would you feel comfortable sharing your DM medical record data in this type of new comprehensive registry? Why/why not
    c. Would you be more likely to participate if you received free genetic testing for your involvement? Why/why not
    d. Would you be more likely to participate if you had 100% personal control of your data, and who your data is shared with in the registry? Why/why not
11. What would you want pharma companies/FDA/clinical trial sites to know?
12. Would you feel comfortable participating in other sorts of studies through MDF? This might include surveys, observations, interviews, etc.
    a. Are there any specific things that come to mind that make you not want to participate?
    b. What about challenges - what might make it hard to participate?
    c. Anything specific that would make you really want to participate?
13. Is there anything else you think we should know that would help MDF determine how they might work to ensure clinical trial success?

**Caregiver Interview Guide**

1. Can you please tell me a little about yourself and your connection to DM?
2. Are you familiar with the differences between natural history studies and clinical drug trials?
   a. Do you see benefits in one over the other?
3. What word(s) comes to mind when you think about the person you care for with DM participating in a clinical trial?
   a. How do you think about the potential risks and rewards of participation?
4. Has the person you care for with DM participated in a clinical drug trial or natural history study before?
   a. If yes, what was that experience like for you?
      i. What went well, if anything?
      ii. What challenges occurred, if anything?
      iii. Is that something you would want them to do again?
   b. If not, why not?
      i. Did you have any conversations with the person about participating?
5. In an ideal world, what would it look like for the person(s) you care for to sign up and participate in a clinical drug trial?
   a. What are the differences between the ideal world and our current reality, if any?
   b. How do we get to the ideal?
6. Does the person you care for participate in MDF’s Family Registry or the National Registry at the University of Rochester?
   a. If yes, why?
      i. What are any benefits you see in participating?
      ii. What are any barriers you see in participating?
   b. If no, why not?
      i. Probe for benefits
      ii. Probe for barriers
7. Comprehensive registries containing information about diagnosis, surveys and assessments completed directly by patients, as well as personal health information and records could greatly improve clinical trials by helping with better participant recruitment and matching. MDF is interested in designing, implementing and managing a comprehensive registry. What comes to mind for you in thinking about the person you care for participating in a registry like the one described?
   a. Would you feel comfortable with a registry that integrates all of the clinical data of the person you care for? Probe why/why not
   b. Would it be beneficial for the healthcare provider providing or entering your relative’s clinical data into a registry FOLLOWING obtaining permission and consent? Probe why/why not
   c. How do you feel about data control and privacy in terms of a registry like this?
8. What could MDF do to help make it easier for the person you care for with DM to get involved in a clinical trial?
9. Would you feel comfortable with the person you care for participating in other sorts of studies through MDF? This might include surveys, observations, interviews, etc.
   a. Why/why not?
   b. What about more caregiver studies?
   c. Are there any barriers to participation that come to mind?
10. Is there anything else you think we should know that would help MDF determine how they might work to ensure clinical trial success?

**Pharmaceutical Focus Group Guide**

1. Does your company currently have an active DM drug development program?

a. If yes, what stage of drug development are you in currently?

2. Is homogeneity of the CTG repeat in people living with DM something you control for in trials?

a. Do you feel that heterogeneity in the participant population could affect trial outcomes?

b. How do you accommodate specific repeat composition information, if at all, as a predictor of individual drug response?

3. How do you, if at all, capture genetic information regarding modifier loci affecting disease progression and drug response?

4. How, if at all, do you control for measuring participants' disease burden and disease duration?

5. Can you please describe what the participant recruitment process looks like for you for DM trials?

a. What does your company’s relationship with patient registries look like?
   i. Do you use them?
   ii. Why/why not?

6. How do you feel about current levels of patient enrollment?

a. What about future levels - do you have any predictions?

b. What can we do to ensure a sufficient number of future patients enrolling?

7. If you could dream up and design a new, state-of-the-art registry as a sophisticated clinical trial resource, what would you want to be included?

a. What patient information would you like to see? (e.g. genetic characterization, individual natural disease history, patient self-reported outcomes, access to research information including potential clinical endpoints)

b. How would you use such a registry?

c. Could you envision assisting with the development of such a registry?

d. Could you envision subscribing to such a registry?

e. Should such a registry also be used to capture P-IV post-market data?

f. Who should own/oversee such a registry and why?

8. How do you feel about current clinical trial outcome measures, such as video hand opening time (vHOT)?

a. Is vHOT something you use or plan to use in a clinical trial?
   i. If yes, why do you plan to use it?
   ii. If not, what do you use instead?

9. How do you approach non-muscular phenotypes in terms of outcome measures?

10. Do you feel myotonia and assessment through vHOT are connected to other clinical endpoints? (For example, CNS, GI, and Cardiac outcomes)

11. How do DM clinical trials compare to the clinical trials of other neuromuscular programs?

12. What do you think about the current duration/endpoint of clinical trials given that DM is a slowly progressing degenerative disease?

13. What does data sharing in the pharmaceutical community look like at this time?

a. Do you currently have access to natural history study data?
   i. If yes, what does that access look like? (probe for cost, time, peer review)
   ii. If not, is that something you would like access to?

14. What does data sharing of your learnings from the FDA process look like at this time?

15. In terms of your/your company’s engagement with the FDA, are there any roadblocks to drug development that you come up against outside of the FDA’s regulatory mandate (i.e. not related to patient and drug safety)?

a. If yes, what structural problems do you see?
b. If you think about a scientific session (= mutual education and working through issues opportunity/session) with the FDA, what would be key topics and outcomes you would like to discuss/see?
   i. Do you think they are specific to your firm or general for the community?

16. From your perspective, what are the top 3 barriers and roadblocks regarding clinical trial readiness today?

a. Probe for site preparedness (staffing, talent, equipment)?

17. How has community input guided your trial design?

a. Do you use Advisory boards, patient panels, ambassadors, or speakers?
   i. When did you begin to engage the community?
   ii. Pre-IND? Trial design phase? Clinical trial phases?

18. Is there anything else you think we should know that would help MDF determine how they might work to ensure clinical trial success?

Question Bank if time allows:

19. What diagnostic changes (advancements) could improve clinical trials?

a. Which ones?

b. How?

20. How do you approach patient education for clinical trials?

a. What challenges, if any, do you encounter?

b. What, if anything, could make this process better?

21. In regards to patient support, (e.g. administering incentives) study/trial sites, is that a concern for you?

a. If yes, please describe any current problems or barriers

b. If not, what is working well for you?

c. What, if anything, could make this process better?

22. From your perspective, is patient apathy a barrier for clinical trials?

a. If yes, what is the impact of this?

b. If not, what is working well for you to work around patient apathy?

c. What, if anything, can MDF do to help avoid problems due to patient apathy?

**Pharmaceutical Survey Questions**

1. How knowledgeable are you regarding the following topics of DM?
   1. Genetics of the disease
   2. Clinical manifestations
   3. Disease burden
   4. Patients’ life impact

*Scale: Level of knowledge:*

*Not knowledgeable*

*Not very knowledge*

*Moderately knowledgeable*

*Knowledgeable*

*Very knowledgeable*

*Unsure or N/A*

2. How satisfied are you with the current speed of clinical trial execution?

3. How satisfied are you with the patient recruitment process?

4. How satisfied are you with the following aspects of your clinical advisors?

a. Knowledge and experience in designing the study

b. Knowledge and experience executing the study protocol

c. Communicating and coordinating between sponsor and CRO

d. Navigating communications with regulatory agencies

e. Successfully resolving regulatory issues

6. Being the voice of the patient
7. Accommodate individual patients’ needs during the trial

*Scale: Level of satisfaction*

*Not satisfied*

*Not very satisfied*

*Moderately satisfied*

*Satisfied*

*Very satisfied*

*Unsure or N/A*

5. How important are the following factors when you are selecting clinical trial sites?

a. Patient availability

b. Access to well-characterized and homogenous patient cohorts?

c. Track record of sites

d. Reputation of clinical site leadership

e. Regulatory environment (e.g. US vs non-US)

f. Cost per patient/study subject

g. Speed at a trial site

h. Patient screening-out rates (in the context of staff and funding constraints)

*Scale: Level of importance*

*Not important*

*Not very important*

*Moderately important*

*Important*

*Very important*

*Unsure or N/A*

6. How ready is your organization to assess non-muscular endpoints?

*Scale: Level of readiness*

*Not ready*

*Not very ready*

*Moderately ready*

*Ready*

*Very ready*

*Unsure or N/A*

7. How ready is the community to assess non-muscular manifestations/endpoints?

*Scale: Level of readiness*

*Not ready*

*Not very ready*

*Moderately ready*

*Ready*

*Very ready*

*Unsure or N/A*

8. Do you feel you are “competing” with other non-DM development programs?

*Yes* /*No* /*Unsure or N/A*

**Clinical Trial Site PI Survey Questions**

1. Which clinical trial/study site do you currently work at? Please include a city/state. If you would prefer to answer anonymously, please write “Anonymous.” However, your responses will be confidential regardless. (open response)
2. What is your role?
3. Has your site ever run interventional clinical trials for Myotonic Dystrophy (DM)?
4. If yes, Is your site currently running interventional clinical trials for Myotonic Dystrophy?
5. If yes, is your site running: (select one)
6. Has your site ever run noninterventional studies (e.g., natural history study, observational studies) for Myotonic Dystrophy (DM)?
7. If yes, Is your site currently running noninterventional studies for Myotonic Dystrophy?
8. If yes, Is your site running: (select one)
   a. One trial for DM
   b. Multiple trials for DM
   c. One trial for DM and other studies for other diseases
   d. Multiple trials for multiple diseases
   e. Unsure
9. How many interventional clinical trials (for any disease) do you anticipate will start in the next 6 months at your site? (text/number response, number rule)
10. How many of those clinical trials do you expect to be for Myotonic Dystrophy? (text/number response, number rule)
11. On average, how many months do you estimate it takes for your site to start a new clinical trial (from site selection to first participant dosed)?

12. Do you currently utilize patient registries, such as the Myotonic Dystrophy Family Registry (MDFR) or University of Rochester (National Registry for DM & FSHD), for DM clinical trial or study recruitment?

13. If yes, what information do you use from registries? Please check all that apply.

a. Genetic information

b. Demographic information

c. Patient location(s)

d. Patient-specific clinical data

e. Other: ____

14. How helpful would a more comprehensive registry with additional information, such as latest patient-reported outcome measures or general clinical data (e.g., labs), be to your site?
15. Considering the time and financial impact of the screening process, in what ways, if at all, do you minimize the chances of a participant failing screening during the enrollment phase of your clinical trial? Please check all that apply.
    a. Participant is known from END-DM1 natural history study
    b. Participant is known from other natural history study
    c. Participant is known from clinic
    d. Participant is registered in registry
    e. Participant initiated contact
    f. Other: ___
16. What level of disease-specific training for staff does your site offer (e.g., training on symptoms or genetics of a disease)?

a. No training

b. Brief overview of the disease for select staff

c. Brief overview of the disease for all relevant staff

d. In-depth training for select staff

e. In-depth training for all relevant staff

f. Unsure

17. How strongly do you agree/disagree with the following statements regarding DM trials? (strongly disagree - strongly agree)

a. Our site does a satisfactory job of explaining how the intervention would potentially affect the disease and symptoms to participants

b. Our site does a satisfactory job of explaining the screening process to trial participants

c. Our site does a satisfactory job of explaining the procedures to trial participants

d. Our site does a satisfactory job of reimbursing trial participants for their travel and accommodations for their visit

e. Our site does a satisfactory job of coordinating care for trial participants during the trial

f. Our site does a satisfactory job of communicating with participants about the end of a trial and what to expect

g. Our site has a high participant retention rate (low drop-out rate)

18. How strongly do you agree/disagree with the following statements regarding DM trials? (strongly disagree - strongly agree)

a. Our staff is knowledgeable about DM

b. Our staff understands the disease impact from the participant perspective

c. Our staff is well-trained in running DM clinical trials

d. Current/future DM clinical trials compete for time and resources with other neuromuscular disease clinical trials at our site

e. Participants have mostly positive experiences participating in clinical trials at our site

f. Our staff prioritizes the participant experience during visits

g. We work to ensure the participant enrollment process is as smooth as possible

h. Our staff maintains a positive relationship with patient advocacy groups, such as Myotonic Dystrophy Foundation (MDF)

19. What do you currently consider to be the top 1-3 challenges in getting clinical trials for myotonic dystrophy up and running? (check up to 3)

a. Staffing (number of staff)

b. Staffing (staff expertise)

c. Finances

d. Bureaucracy (i.e., dealing with regulations)

e. Belief in the success of the intervention

f. Participant recruitment

g. Participant enrollment

h. Participant retention

i. Participant education

j. Equipment (e.g., purchasing, set up, function)

k. Extensiveness of the assessment protocols

l. Competing with other neuromuscular disease clinical trials

m. Communication with sponsors

n. Other _____

o. Unsure

20. Does your site have the ability to provide drug infusions (e.g., for future interventional studies)?

21. If yes, where are infusions currently being given?

22. If yes, what type of medical professionals provide your infusions?

23. Are there barriers to providing infusions at your site? Please check all that apply.

a. Time

b. Space

c. Personnel

d. Costs

e. Other competing therapies

f. None

g. Other: ___

h. Unsure

24. How involved are you in the design/development of the clinical trial protocol for DM trials?

a. Very involved

b. Somewhat involved

c. Not involved

d. It depends on the sponsor

e. Unsure

25. Do you (or do you plan to) control for homogeneity of the CTG repeat in people living with DM in trials (beyond inclusion/exclusion criteria)?

26. Do you (or do you plan to) look at CTG repeat length as a variable for treatment outcomes?

27. Do you (or do you plan to) look at CTG repeat composition (e.g. repeat disruption) as a variable for treatment outcomes?

28. How likely do you think repeat composition and/or modifier loci could play a role in disease manifestation and disease severity? (scale: Very likely to very unlikely)

29. Do you (or do you plan) to look at other modifier loci (e.g., MSH3, CELF1, MLH1/MLH3) and how they would impact drug response and/or subcategorize participants?

30. How prepared do you feel your site is for the upcoming DM clinical trials?

a. Very prepared: ready for multiple (3+) trials to start in the next year or so

b. Prepared: ready for 1-2 trials to start in the next year or so

c. Somewhat prepared: we can be ready for multiple trials to start, but in the next 3-5 years

d. Not so prepared: we need time to prepare for 1-2 trials to start within the next 3-5 years

e. Other: ___

f. Unsure

31. Has your site been affected by recent policy and funding cuts at federal health agencies?

32. If yes, will these funding cuts affect your ability to conduct DM trials or studies?

33. If yes, how? (open text)

34. Please rate your agreement/disagreement with the following statements about people living with DM. (strongly disagree - strongly agree)

a. People living with DM, in general, have a reasonable understanding of the underlying biology of the disease.

b. People living with DM, in general, have a reasonable understanding of the clinical trial process, including phases and placebo groups

c. People living with DM, in general, are excited about upcoming clinical drug trials

d. People living with DM, in general, are prepared to participate in upcoming clinical drug trials

e. Patient recruitment for upcoming clinical drug trials is not a concern–we have enough participants that meet eligibility criteria

f. The DM community has sufficient knowledge about gene therapies

g. People living with DM, in general, have sufficient knowledge to distinguish between gene therapy and regular infusion-based application modalities

h. The DM community is aware of ongoing or future clinical trials for treatments

35. Please rate your agreement/disagreement with the following statements about DM as a disease (strongly disagree - strongly agree)

a. DM is difficult to diagnose because of high variability in clinical presentation

b. High variability in DM makes it difficult to create inclusion/exclusion criteria for trials

c. DM requires special staff and care, such as physical therapists, at trial/study sites

d. The symptoms of DM, such as apathy, can be an issue for running clinical trials

36. What endpoints for neuromuscular diseases (such as DM) is your facility currently equipped to measure? (check all that apply)

a. Video Hand Opening Time (vHOT)

b. Muscle strength/function (e.g., hand-grip, 10m run-walk, other)

c. Muscle biopsy and related molecular tests (e.g., splicing assessments)

d. Disease impact for participants through the DM1-Activ-c assessment

e. Disease impact for participants through the Myotonic Dystrophy Health Index (MDHI) assessment

f. Heart function: Please describe ___

g. Cognitive function: Please describe _______

h. Imaging-based analyses (e.g., MRI, CT): Please describe _____

i. GI function: Please describe _____

j. Other: ____

k. Unsure

37. Do you think that current clinical trials for DM capture the relevant endpoints for this disease?

a. Yes, current endpoints are satisfactory

b. No, we need to be looking at more endpoints

c. No, we are looking at the wrong endpoints currently

d. Unsure

38. If no, what additional endpoints do you think would be helpful for DM? (text box)

39. Please rate your agreement/disagreement with the following statements about current DM endpoints for clinical trials. (strongly disagree - strongly agree)

a. Current endpoints for DM are meaningful and relevant for the majority of DM patients.

b. Current endpoints accurately capture the disease impact that the majority of patients experience in their daily lives.

c. Measurements of current endpoints are sufficiently standardized.

d. We need more research on what endpoints are possible for DM trials.

e. The research and clinical community should have a structured forum (i.e., a workgroup) for the development of relevant and emerging endpoints for DM clinical trials.

f. Patients provide sufficient input for trial design regarding meaningful trial endpoints.

g. Pharmaceutical companies are motivated and willing to look for other DM endpoints.

h. The FDA and other regulatory bodies are motivated and willing to look for other DM endpoints.

40. Which regulatory bodies do you interact with for DM clinical trials? Please check all that apply.

a. FDA

b. EMA

c. Health Canada

d. Other: ___

41. Please rate your agreement/disagreement with the following statements about regulators for DM clinical trials (e.g., FDA, EMA, Health Canada). (strongly disagree - strongly agree)

a. Regulators have sufficient knowledge about DM as a disease.

b. Regulators are satisfied with the current endpoints for DM.

c. Regulators are satisfied with current knowledge regarding the long-term natural history of the disease.

d. Regulators correctly balance potential risks and benefits for new drug treatments.

e. Regulators should be less cautious when it comes to the risks of drugs.

f. Regulators support vHOT as a meaningful outcome for DM trials.

g. Regulators understand the burden that patients with DM face on a daily basis.

h. Regulators are satisfied that current endpoints reflect patients' impacts and experiences.

i. It is harder to get a DM study approved in the US than it is in other countries.

42. Please rate your agreement/disagreement with the following statements. (strongly disagree - strongly agree)

a. I have access to the natural history study data that is currently available.

b. The current two-year END-DM1 natural history study is sufficient for clinical trials.

c. We need more long-term natural history data for DM.

d. Everyone should be able to access natural history study data for DM for free.

e. Data sharing is a problem in the DM research community.

43. Would you be interested in participating in a structured forum with regulators to share best practices in clinical trials?

44. Would you be open to attending and/or sharing your perspectives in a meeting with other researchers about DM endpoints?

45. What other support(s) might people living with DM need when they come in for clinical trials (interventional and noninterventional)? (open text)

46. From your perspective, what are 1-3 things that a patient advocacy group, such as MDF, could do to maximize the success of clinical trials (interventional and noninterventional) for DM? (open text)

47. From your perspective, what is the biggest challenge for participants during clinical trials (interventional and noninterventional)? (open text)

48. If MDF were able to hold a scientific session with the FDA about clinical trials in myotonic dystrophy, what 1-3 questions would you want to ask? (open text)

49. What would be the desired outcomes of a scientific session with the FDA? (open text)

50. How does drug development for DM compare to other rare diseases and/or neuromuscular disorders? (open text)

51. From your perspective, what are the 1-3 biggest barriers to clinical **interventional** trial readiness for the DM community (including people living with DM, caregivers, pharmaceutical partners, and trial sites)? (open text)

**Clinical Trial Site Coordinator Survey Questions**

1. Which clinical trial/study site do you currently work at? Please include a city/state. If you would prefer to answer anonymously, please write “Anonymous.” However, your responses will be confidential regardless. (open response)
2. What is your role?
3. Has your site ever run interventional clinical trials for Myotonic Dystrophy (DM)?
4. If yes, Is your site currently running interventional clinical trials for Myotonic Dystrophy?
5. If yes, is your site running: (select one)
6. Has your site ever run noninterventional studies (e.g., natural history study, observational studies) for Myotonic Dystrophy (DM)?
7. If yes, Is your site currently running noninterventional studies for Myotonic Dystrophy?
8. If yes, Is your site running: (select one)
   a. One trial for DM
   b. Multiple trials for DM
   c. One trial for DM and other studies for other diseases
   d. Multiple trials for multiple diseases
   e. Unsure
9. How many interventional clinical trials (for any disease) do you anticipate will start in the next 6 months at your site? (text/number response, number rule)
10. How many of those clinical trials do you expect to be for Myotonic Dystrophy? (text/number response, number rule)
11. On average, how many months do you estimate it takes for your site to start a new clinical trial (from site selection to first participant dosed)?

12. How many clinical research coordinators does your site typically have?

a. One coordinator working on multiple trials

b. One coordinator per trial

c. Many coordinators for each trial

d. It depends on the trial (there is no “typical” case)

e. Unsure

13. Do you currently utilize patient registries, such as the Myotonic Dystrophy Family Registry (MDFR) or University of Rochester (National Registry for DM & FSHD), for DM clinical trial or study recruitment?

14. If yes, what information do you use from registries? Please check all that apply.

a. Genetic information

b. Demographic information

c. Patient location(s)

d. Patient-specific clinical data

e. Other: ____

15. Is your site responsible for determining participant eligibility for the trial based on inclusion/exclusion criteria?

16. If yes, what happens when a participant is ineligible? Please check all that apply.

a. Direct follow-up call with the participant

b. Email or written communication to the participant

c. Explanation of eligibility criteria

d. Explanation of why the participant is ineligible

e. None of the above

f. Other: ____

g. Unsure

17. If yes, On average, what percentage of participants screened end up being enrolled in the actual trial? Use your best guess based on your experience.

18. Considering the time and financial impact of the screening process, in what ways, if at all, do you minimize the chances of a participant failing screening during the enrollment phase of your clinical trial? Please check all that apply.

a. Participant is known from END-DM1 natural history study

b. Participant is known from other natural history study

c. Participant is known from clinic

d. Participant is registered in registry

e. Participant initiated contact

f. Other: ___

19. What level of disease-specific training for staff does your site offer (e.g., training on symptoms or genetics of a disease)?

a. No training

b. Brief overview of the disease for select staff

c. Brief overview of the disease for all relevant staff

d. In-depth training for select staff

e. In-depth training for all relevant staff

f. Unsure

20. What accessibility accommodations does your site offer? Please check all that apply.

a. Handicapped parking spaces

b. Wheelchair ramps

c. Accessibility-designed spaces (e.g. wheelchair-height countertops, wide hallways, extra large exam rooms)

d. Elevators

e. Parking/valet services with accessible vehicles

f. Outpatient laboratory option

g. None of the above

h. Other: ____

i. Unsure

21. Does your site coordinate participant travel?

22. If yes, who is responsible for booking participant travel, including day-of transit?

23. If yes, who is responsible for booking participant hotel accommodations?

24. What specific accommodations do you provide for participants with myotonic dystrophy? Please check all that apply.

a. Later start times (i.e., after 10 am)

b. Shorter days/spread out appointments

c. Multiple follow-ups and reminders about appointments

d. Breaks during the day

e. Accessible transportation vehicles

f. Child/family care during appointments

g. Caregiver supports during appointments

h. Accessible or family bathrooms on each floor

i. Wheelchair-friendly building

j. Designated coordinator/point of contact

k. Storage space for medical equipment/luggage

l. Hotel partnership nearby

m. Reimbursement for lodging the night before and after appointment

n. On-site physical therapist

o. None of the above

p. Other: ____

q. Unsure

25. Does your site offer home visits or local clinic options for certain appointments, such as blood draws or vitals? Please check all that apply.

26. Does your site provide any support/programs for caregivers who attend the trial appointments?

27. If yes, please describe: (open text)

28. Does your site have the ability to provide drug infusions (e.g., for future interventional studies)?

29. If yes, where are infusions currently being given?

30. If yes, what type of medical professionals provide your infusions?

31. Does your site coordinate visit reimbursements for participants?

32. If yes or sometimes, what form of incentive payment or reimbursement do you provide? Please check all that apply.

a. Direct deposit

b. Prepaid debit card via mail

c. Prepaid debit card via email

d. Check on-site or via mail

e. Cash on-site or via mail

f. It depends on the trial/sponsor

g. None of the above

h. Other: ____

i. Unsure

33. How strongly do you agree/disagree with the following statements regarding DM trials? (strongly disagree - strongly agree)

a. Our site does a satisfactory job of explaining how the intervention would potentially affect the disease and symptoms to participants

b. Our site does a satisfactory job of explaining the screening process to trial participants

c. Our site does a satisfactory job of explaining the procedures to trial participants

d. Our site does a satisfactory job of reimbursing trial participants for their travel and accommodations for their visit

e. Our site does a satisfactory job of coordinating care for trial participants during the trial

f. Our site does a satisfactory job of communicating with participants about the end of a trial and what to expect

g. Our site has a high participant retention rate (low drop-out rate)

34. How strongly do you agree/disagree with the following statements regarding DM trials? (strongly disagree - strongly agree)

1. Our staff is knowledgeable about DM

2. Our staff understands the disease impact from the participant perspective

3. Our staff is well-trained in running DM clinical trials

4. Current/future DM clinical trials compete for time and resources with other neuromuscular disease clinical trials at our site

5. Participants have mostly positive experiences participating in clinical trials at our site

6. Our staff prioritizes the participant experience during visits

7. We work to ensure the participant enrollment process is as smooth as possible

8. Our staff maintains a positive relationship with patient advocacy groups, such as Myotonic Dystrophy Foundation (MDF)

35. What do you currently consider to be the top 1-3 challenges in getting clinical trials for myotonic dystrophy up and running? (check up to 3)

a. Staffing (number of staff)

b. Staffing (staff expertise)

c. Finances

d. Bureaucracy (i.e., dealing with regulations)

e. Belief in the success of the intervention

f. Participant recruitment

g. Participant enrollment

h. Participant retention

i. Participant education

j. Equipment (e.g., purchasing, set up, function)

k. Extensiveness of the assessment protocols

l. Competing with other neuromuscular disease clinical trials

m. Communication with sponsors

n. Other _____

o. Unsure

36. What other support(s) might people living with DM need when they come in for clinical trials (interventional and noninterventional)? (open text)

37. From your perspective, what are 1-3 things that a patient advocacy group, such as MDF, could do to maximize the success of clinical trials (interventional and noninterventional) for DM? (open text)

38. From your perspective, what is the biggest challenge for participants during clinical trials (interventional and noninterventional)? (open text)

39. From your perspective, what are the 1-3 biggest barriers to clinical **interventional** trial readiness for the DM community (including people living with DM, caregivers, pharmaceutical partners, and trial sites)? (open text)
